# Diverse inflammatory threats modulate astrocytes Ca^2+^ signaling via connexin43 hemichannels in organotypic spinal slices

**DOI:** 10.1186/s13041-021-00868-6

**Published:** 2021-10-25

**Authors:** Giulia Panattoni, Roberta Amoriello, Christian Memo, Agnes Thalhammer, Clara Ballerini, Laura Ballerini

**Affiliations:** 1 International School for Advanced Studies (SISSA/ISAS), 34136 Trieste, Italy; 2 Dipartimento di Medicina Sperimentale e Clinica, University of Florence, 50139 Florence, Italy

**Keywords:** Pro-inflammatory cytokines, LPS, Live imaging, Neuroinflammation, Gap junctions, Hemichannels, Spinal neurons, Immune resident cells

## Abstract

Neuroinflammation is an escalation factor shared by a vast range of central nervous system (CNS) pathologies, from neurodegenerative diseases to neuropsychiatric disorders. CNS immune status emerges by the integration of the responses of resident and not resident cells, leading to alterations in neural circuits functions. To explore spinal cord astrocyte reactivity to inflammatory threats we focused our study on the effects of local inflammation in a controlled micro-environment, the organotypic spinal slices, developed from the spinal cord of mouse embryos. These organ cultures represent a complex in vitro model where sensory-motor cytoarchitecture, synaptic properties and spinal cord resident cells, are retained in a 3D fashion and we recently exploit these cultures to model two diverse immune conditions in the CNS, involving different inflammatory networks and products. Here, we specifically focus on the tuning of calcium signaling in astrocytes by these diverse types of inflammation and we investigate the mechanisms which modulate intracellular calcium release and its spreading among astrocytes in the inflamed environment. Organotypic spinal cord slices are cultured for two or three weeks in vitro (WIV) and exposed for 6 h to a cocktail of cytokines (CKs), composed by tumor necrosis factor alpha (TNF-α), interleukin-1 beta (IL-1 β) and granulocyte macrophage-colony stimulating factor (GM-CSF), or to lipopolysaccharide (LPS). By live calcium imaging of the ventral horn, we document an increase in active astrocytes and in the occurrence of spontaneous calcium oscillations displayed by these cells when exposed to each inflammatory threat. Through several pharmacological treatments, we demonstrate that intracellular calcium sources and the activation of connexin 43 (Cx43) hemichannels have a pivotal role in increasing calcium intercellular communication in both CKs and LPS conditions, while the Cx43 gap junction communication is apparently reduced by the inflammatory treatments.

**Supplementary Information:**

The online version contains supplementary material available at 10.1186/s13041-021-00868-6.

## Introduction

Neuroinflammation is a characterizing feature occurring in and contributing to CNS pathologies such as amyotrophic lateral sclerosis (ALS) and multiple sclerosis (MS) [1, 2]. In the CNS, successful inflammatory responses exert a protective homeostatic action, on the contrary, protracted tissue reactivity sustains unregulated cytokine/chemokine release and chronic inflammation, which has been proposed as a major cause of disease progression [3]. In the last decade, several studies documented, associated to neuroinflammatory processes, the appearance of synaptic dysfunction, i.e. synaptopathy [4, 5]. Indeed, the emergent activity of neural circuits may be altered both acutely and chronically by inflammatory milieus activating intricate signaling pathways, orchestrated by various cell phenotypes, ultimately responsible for intercellular communication and contributing to the propagation of the inflammatory damage in the CNS. In this picture, astrocytes, the key cellular partners to neurons, play both beneficial roles, such as recovery of extracellular ionic homeostasis limiting inflammation [6] and deleterious ones, such as hypertrophy with increased astrogliosis [7, 8]. Knowing how astrocytes perform such signals might allow to selectively promote their beneficial functions and inhibit the adverse ones in diseased CNS.

We recently investigated the effects on spinal synaptic outputs of different inflammatory threats, focusing on the consequences of local inflammation in a controlled micro-environment: the organotypic slice cultures developed from the embryonic mouse spinal cord explants [9–11]. Core features of this in vitro model are the 3D organization of spinal cord resident cells and the preserved sensory-motor cytoarchitecture [11–13]. This model allows the study of spinal tissue alterations induced by inflammation, addressing the role of resident cells: neuronal and not neuronal populations. By the use of spinal cord explant cultures, we reported the ability of two diverse immune conditions to improve network excitability by specific synaptic mechanisms [11].

In the present study, we exploit spinal slice cultures to explore astrocyte recruitment upon exposure (6 h) to pro-inflammatory CKs cocktail (TNF-α, IL-1β and GM-CSF [4, 14, 15]) or to LPS, a potent trigger of cytokine release [16, 17] largely adopted to elucidate the mechanisms of brain inflammation. In our previous experiments, CKs and LPS treatments mediated an increase in cytokines and chemokines production, although differently regulating the morphology of resident neuroglia, suggestive of diverse activation states [11]. In reactive tissues, astrocytes can be neuroprotective or neurotoxic, depending on the context, and Ca^2+^ signaling has a key role in these processes [18]. Reactive astrocytes are known to increase dynamic Ca^2+^ signals, shown to be crucial to intracellular signaling and intercellular communication [19]. Such calcium dynamics were reported to vary in distinct pathological models and regions, indicating that aberrant Ca^2+^ signals may depend on the context conditions [18].

To examine astrocytes responses to inflammation, we monitor live Ca^2+^ signals within the spinal cord cultured microcircuits. We focus on astrocytes located in the ventral horn within pre-motor networks and we compared their calcium signaling when activated by CKs or LPS. We document the timing and appearance of intracellular calcium oscillations upon CKs or LPS exposure, such astrocyte episodes are generated by each treatment independently from the ongoing synaptic activity. We further show, by pharmacological treatments, that CKs and LPS induce calcium release from endoplasmic reticulum, mitochondria and that astrocyte pro-inflammatory activation as well as cytokines and chemokines production, are tuned by gap junctions (GJs) and hemichannels (HCs) regulation.

## Results

### Sulforhodamine-positive glial cells display slow spontaneous Ca^2+^ activity

**Fig. 1:**
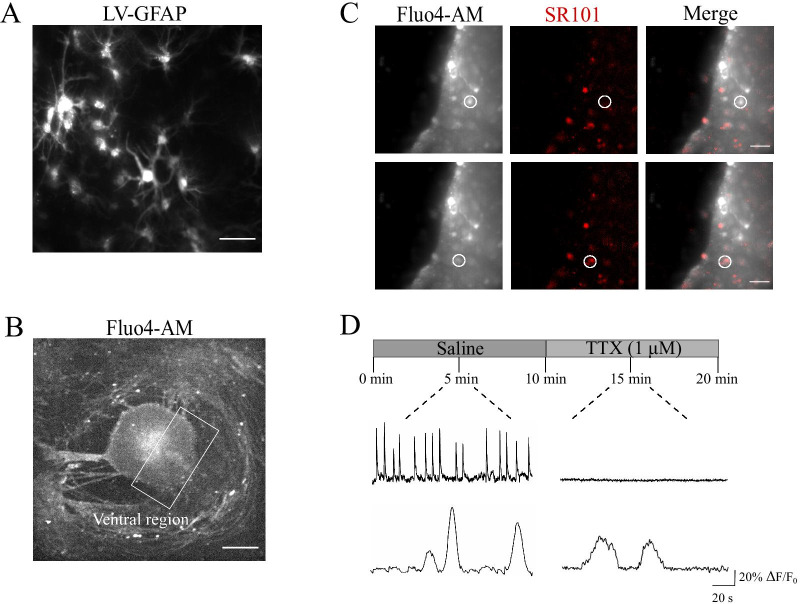
SR101-positive glial cells display spontaneous, slow Ca^2+^ oscillations.** A** Representative images of GFAP-LV vectors visualizing astrocytes in the ventral-horn of the organotypic spinal slice. Scale bar 50 µm. **B** Representative fluorescent image at low magnification (4 ×) of a spinal organotypic slice loaded with the calcium dye Fluo4-AM (4 µM). The ventral region (white frame) is identified by the ventral fissure. Scale bar, 500 µm. **C** CCD-camera snapshots visualize cells located at the border of the ventral region and loaded with Fluo4-AM, in grey (left), and with SR101, in red (middle). Merged images on the right. Scale bars, 50 µm. **D** Top, representative fluorescent tracing of spontaneous neuronal Ca^2+^ activity, prior and after 1 µM tetrodotoxin (TTX), recorded from SR101 negative cell (same as in **C**). Bottom, representative fluorescent tracing of astrocytes Ca^2+^ oscillations, before and after the application of TTX, recorded from SR101 positive cell (same as in **C**)

The presence of GFAP-positive astrocytes has long been described in cultured spinal explants ventral horns [11–13], as confirmed by Fig. 1A, where numerous astrocytes are visualized within the ventral area of a spinal organotypic culture after 2 WIV. We labeled by fluorescent dye Fluo-4 AM (see “Methods”) cells in organotypic spinal cord and dorsal root ganglia (DRG) co-cultures to simultaneously visualize within the sampled area (visual field 330 × 330 μm^2^, Fig. 1B) of the ventral horn pre-motor circuit [11], neuronal and glial cells calcium signaling. To reliably identify astrocytes for physiological measurements of calcium dynamics, we took advantage of a widely used astrocyte marker, sulforhodamine (SR101; 1 µM, Fig. 1C) [20–22] enabling the detection of living astrocytes during ex vivo calcium imaging experiments, in combination with the calcium dye Fluo-4 AM. As shown in Fig. 1C (top panels; white circles) and in the corresponding sample tracings of spontaneous fluorescent recordings in Fig. 1D (top traces), the imaging of visually identified small neurons close to the ventral fissure [23] resulted in SR101-negative cells highly active in control saline solution, with fast (4.12 ± 0.63 s duration; n = 14 cells) calcium episodes which were silenced after application of tetrodotoxin (TTX, 1 µM; fast voltage-gated sodium channel blocker to remove action potentials [23]; Fig. 1D), confirming their neuronal identity. Differently, closely located SR101-positive cells (Fig. 1C, bottom panels; white circles) were spontaneously less active (Fig. 1D, bottom tracings) and typically displayed slow (21.6 ± 1.55 s duration; n = 42 cells) and rare episodes of spontaneous activity, which were resistant to TTX application and allowed identifying glial cells calcium dynamics. In these conditions, we never detected short-lasting Ca^2+^ transients (less than 8 s; Fig. 1D) [24, 25]. To further strengthen the astrocytic origin of the calcium events detected in TTX, in a separate set of experiments we used the genetically-encoded calcium dye GCaMP6f exclusively expressed in astrocytes [26] (n = 4) and we confirmed the occurrence of low-frequency and irregular calcium transients which were not affected by TTX (Additional file 1: Fig. S1A). In all subsequent experiments we used the spatial location, the slow kinetic and the TTX resistance to identify glial calcium signaling.

### CKs and LPS treatments affect Ca^2+^ transients in organotypic spinal slices

**Fig. 2:**
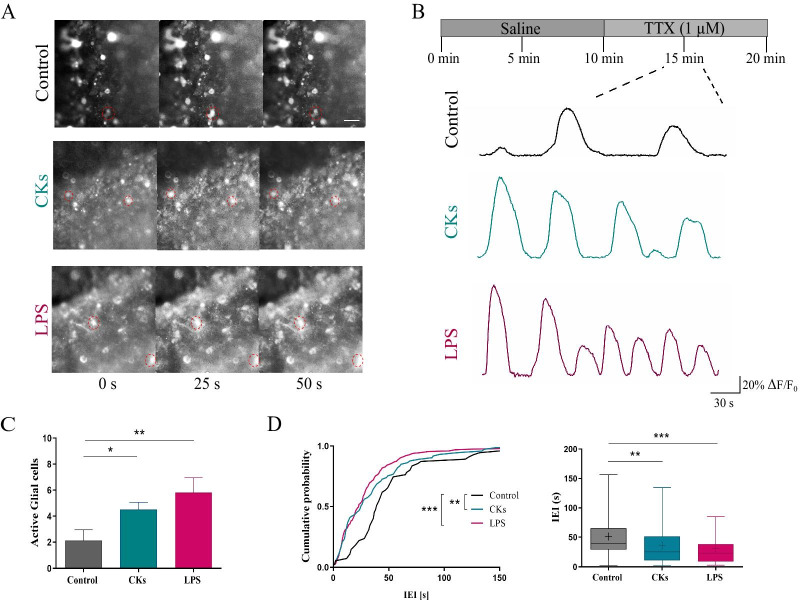
CKs and LPS enhance spinal astrocytes calcium dynamics. **A** Representative snapshots (40 × magnification) of the ventral area of organotypic spinal slices loaded with Fluo-4 AM (4 µM); frames were taken at variable time intervals (0, 25 and 50 s) in three different experimental conditions (Control, CKs and LPS). Scale bar 50 µm. **B** Representative fluorescent tracings depicting glial cells calcium oscillations in Control (black), CKs and LPS (6H, blue and purple, respectively), all recorded in the presence of TTX. **C** The bar plot summarizes the number of spontaneously active glial cells/field. **P < 0.01 and *P < 0.05, one-way ANOVA. **D** Cumulative distributions and box plot quantify the IEIs (s) of the recorded calcium activity in all conditions. ***P < 0.001 and **P < 0.01, in the cumulative plot Kolmogorov–Smirnov test and in the Box plot, Kruskal–Wallis test

To investigate the ability of inflammation to impact calcium dynamics in glial cells, organotypic spinal explants were treated (6H, see “Methods”) with two danger signals triggering different, although well characterized, inflammatory states in these cultures [2, 3]: a pro-inflammatory cocktail of CK (10 ng/mL; TNF-α, IL-1 β and GM-CSF) [11, 13] and the LPS (1 µg/mL) [11, 17] stimuli. First, we monitored ventral neurons calcium signaling and we compared activity from neurons prior (Control; n = 8) and after CKs (n = 7) or LPS (n = 7) treatments. In the Additional file 1: Fig. S1B, snapshots of neurons stained with membrane permeable dye Fluo-4 AM are visualized in all conditions. Neurons, in CKs and LPS treated slices (Additional file 1: Fig. S1C) displayed a significant increase in the frequency of basal calcium oscillations, when compared to Control (0.24 ± 0.01 Hz Control, 0.31 ± 0.02 Hz CKs and 0.39 ± 0.03 Hz LPS; n = 7 each; ***P < 0.001 Control vs LPS and **P = 0.0052 Control vs CKs, Kruskal–Wallis test; box plot in Additional file 1: Fig. S1C). This boost in activity was accompanied by high synchronization among correlated pairs of cells simultaneously recorded (see “Methods” and Additional file 2: Fig. S2A). Such an enhanced calcium dynamics probably reflect the high degree of spontaneous synaptic activity typical of spinal cord preparations when inflammatory states are activated [11, 13] and was not further examined. Calcium signaling due to synaptic activity was removed by TTX application, as shown in Fig. 1D, and we next explored glial cells activity and in particular how astrocytes respond to the localized inflammation, since it is known that also astrocytes display spontaneous calcium signaling [27–29]. Calcium dynamics in glial cells was measured always in the presence of TTX and in Control slices (n = 8) was characterized by the sporadic (on average 2.1 ± 0.29 active cells per recorded field) appearance of slow oscillations, quantified by inter event intervals (IEIs) of 51.3 ± 5.4 s (Fig. 2 A-D). Upon CKs (n = 7) and LPS (n = 7) treatments, an increased number of active cells per recorded field was detected in respect to Control (4.5 ± 0.56 active cells in CKs and 5.8 ± 1.16 active cells in LPS; **P = 0.003 Control vs LPS and *P = 0.04 Control vs CKs, one-way ANOVA test; bar plot in Fig. 2C) displaying calcium oscillations characterized by a significant reduction in the IEIs when compared to the Control ones (36.5 ± 4.3 s in CKs and 31.1 ± 2.5 s in LPS; ***P < 0.001 Control vs LPS and **P = 0.005 Control vs CKs, Kruskal–Wallis test; cumulative probability plot and box plot in Fig. 2 D). Glial cell calcium oscillations were also analyzed for their synchronicity among simultaneously recorded cells. To this aim we measured in each slice (n = 3 each, Control, CKs and LPS) the activity of distant (see “Methods”; Additional file 2: Fig. S2B) pairs of active glial cells (n = 6, 20 and 22 cell pairs in Control, CKs and LPS). Increase active cells during inflammatory threats, regardless the triggering by CKs or LPS, were significantly less synchronized when compared to Control ones (0.05 ± 0.01 pair p-value Control; 0.21 ± 0.02 pair p-value CKs 6H; 0.33 ± 0.02 pair p-value LPS 6H, ***P < 0.001 Control vs LPS and **P = 0.0025 Control vs CKs, one-way ANOVA test; χ^2^ = 7.73, *P = 0.02, Fisher’s exact test; Additional file 2: Fig. S2B). Interestingly, in physiological states, GJs are usually responsible for the synchronization of glial calcium activity [30–32], which is often reduced by diverse pathological conditions [33].

Despite the similar response in terms of glial calcium dynamics, from the morphological point of view, CKs and LPS treatments differently affected the appearance of resident astrocytes and microglia, with opposite changes in Iba1 positive microglia dendritic arborizations (quantified by the transformation index; Additional file 1: Fig. S1D and box plot in E (bottom); with average index values 5.2 ± 2.4 Control, n = 4; 2.2 ± 1.7 CKs, n = 4; 13.2 ± 2.4 LPS, n = 4; ***P < 0.001 Control vs LPS 6H and *P = 0.03 Control vs CKs, Kruskal–Wallis test), and in GFAP intensity enhancement, which was typically milder in LPS treatments in respect to CKs (Additional file 1: Fig. S1D and box plot in E, top; n = 10, 11, 10, Control, CKs and LPS respectively; in a.u. 799.9 ± 374.1 Control; 2312 ± 318.1 CKs and 1404 ± 586.5 LPS; **** *P* ≤ 0.0001 Control vs CKs and CKs vs LPS, and * *P* = 0.0112 Control vs LPS, one way ANOVA). These results are in agreement and confirm our previous observations [11].

### Intracellular stores, gap-junction and hemi-channels dependence of glial calcium signals

**Fig. 3:**
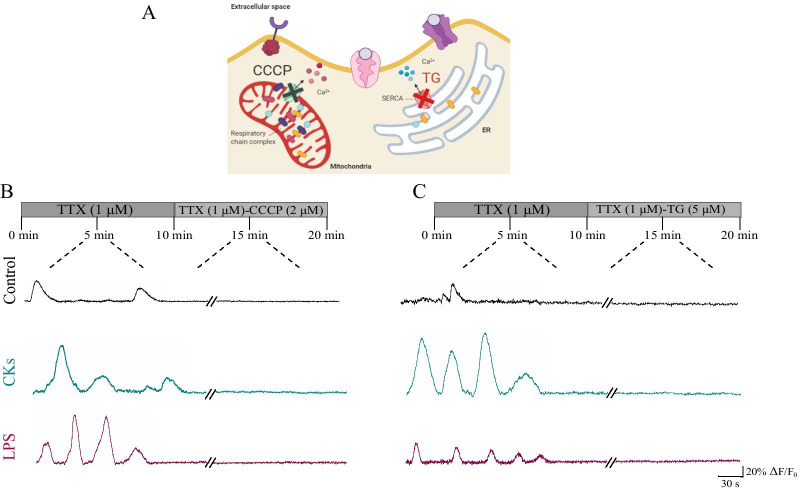
Intracellular calcium sources sustain calcium dynamics in astrocytes.** A** Sketch of the intracellular calcium sources targeted by the blockers used. **B** Fluorescent tracings from representative glial cells prior and after CCCP mitochondrial uncoupler administration in Control (black), CKs and LPS (blue and purple, respectively). **C** Fluorescent tracings from representative glial cells prior and after Thapsigargin (TG) administration in Control (black), CKs and LPS (blue and purple, respectively). All recordings were performed in the presence of TTX. Note the removal of calcium activity by each treatment in each condition

In order to explore the nature of the observed oscillatory patterns, in Control as well as in CKs and LPS, we investigated the dependence on intracellular calcium stores of these activities. We assessed the contribution of internal Ca^2+^ sources from the mitochondria and the endoplasmic reticulum (sketched in Fig. 3A). To explore the mitochondrial role, we used the protonophore carbonyl cyanide 3-chlorophenylhydrazone (CCCP, 2 µM) [23] to dissipate the proton gradient across the inner mitochondrial membrane and disrupt the Ca^2+^ uptake. After CCCP application, Ca^2+^ transients completely disappeared, this response did not differ among the three experimental conditions (n = 8, 7 and 5, Control, CKs and LPS, respectively; Fig. 3B). Next, we explored the contribution of intracellular Ca^2+^ source, namely the endoplasmic reticulum, to the calcium events [23]. Application of the non-competitive inhibitor of the sarco/endoplasmic reticulum Ca^2+^ ATPase (SERCA) thapsigargin (TG, 5 µM) blocked all calcium transients in the three conditions (n = 8, 9 and 5, Control, CKs and LPS, respectively; Fig. 3C). These results suggest that spontaneous intracellular Ca^2+^ transients in single astrocytes in Control as well as in inflamed spinal tissues strongly rely on intracellular calcium sources.

In tissue cultures, Ca^2+^ events can propagate among a network of astrocytes via GJs [34], allowing synchronization of the calcium activity. GJs are formed by the docking of two HCs and unapposed HCs, not assembled into GJs, are present in the plasma membrane. Non-junctional HCs may be activated in response to inflammation stimuli [35], thereby allowing ionic and molecular exchange between the intra- and extracellular environment. In the absence of neurotransmitter activation, cultured explant in CKs or LPS did not show propagated calcium signals or synchronization among astrocytes, however astroglial cells usually express connexins that support long-range communication. GJs and connexins are also known to have an enhanced turnover in the inflamed tissues [31, 32, 36] while HCs in non-junctional membrane and GJs are oppositely regulated by various conditions [37].

**Fig. 4:**
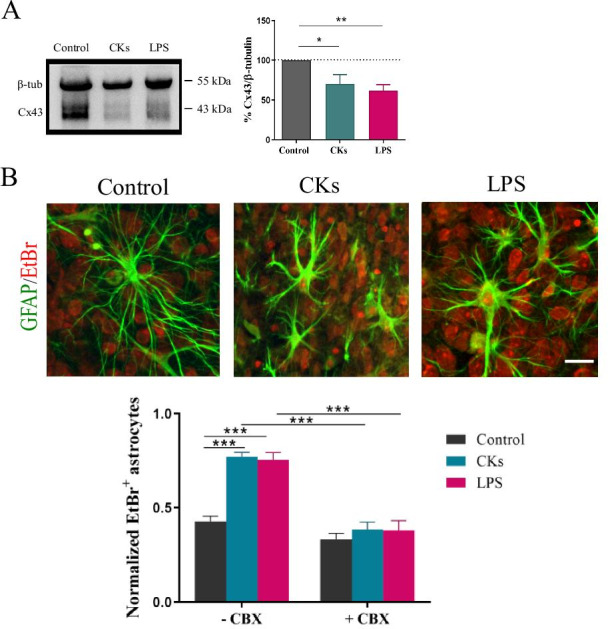
Cx43 GJs and HCs role in the increased calcium dynamics in CKs and LPS.** A** Western blot experiments (left) and analysis (bar plot, right) for the protein Cx43 in Control, CKs and LPS treated slices. Values are expressed as % of Control. **P < 0.01 and *P < 0.05, one-way ANOVA. **B** Representative high magnification (40 ×) confocal images of EtBr uptake (30 min, in red) in astrocytes (GFAP, in green) induced by CKs and LPS treatments, scale bar 25 µm. EtBr uptake is quantified in the bar plot, normalized to the total number of astrocytes/field, in the absence or in the presence of carbenoxolone (CBX, 200 µM) pre-incubation (30 min). ***P < 0.001, one-way ANOVA

Since both GJs and HCs in astrocytes in culture are mainly composed by Cx43 protein [30, 38] to address potential changes in the expression of Cx43 we used immunoblotting analysis under the three different experimental conditions. The western blot (WB) in Fig. 4A shows the protein bands in the three culture groups (n = 8, 7 and 8, Control, CKs and LPS, respectively) and we observed that CKs and LPS treatments significantly (**P = 0.004 Control vs LPS and *P = 0.03 Control vs CKs, one-way ANOVA test; bar plot in Fig. 4A) reduced the expression of Cx43, in respect to Control, in agreement with previous works [36, 39]. It has been shown that proinflammatory CKs released by activated microglia, inhibit GJs mediated by Cx43, whereas opening HCs, a pathway enabling release of active molecules [39]. In the presence of reduced Cx43 expression, CKs and LPS might still enhance calcium activity in astrocytes by the opening of HCs.

The WB-blot analysis of Cx43 expression is in accordance with the functional evidence of desynchronization of calcium events in astrocytes detected upon inflammatory states, both results being supportive of a reduced GJ communication in the presence of pro-inflammatory stimuli, however HCs opening cannot be excluded [37]. We used the fluorescent dye ethidium bromide (EtBr), a tool adopted to investigate HCs permeability [39–41], to assess the presence of changes in HC activity upon CKs and LPS exposures. Control and treated slices (n = 5, 8, 7, control, CKs and LPS, respectively) were incubated (30 min) with EtBr (5 µM), as shown in Fig. 4B and EtBr positive glial cells were quantified in the bar plot (normalized to GFAP-positive cells: 0.41 ± 0.03 Control; 0.77 ± 0.02 CKs; 0.76 ± 0.04 LPS; *** *P* < 0.0001, one-way ANOVA test). We used carbenoxolone (CBX 200 µM, 30 min), a well-known GJs uncoupler [23, 41, 42] and a blocker of active HCs [43] to test the functional role of HCs in the observed dye uptake. When Control and treated slices were pre-incubated (n = 5, 9, 5, Control, CKs and LPS, respectively) with CBX, the astrocyte dye-uptake in treated slices was significantly reduced (0.36 ± 0.04 CKs + CBX; 0.36 ± 0.05 LPS + CBX; *** *P* < 0.0001, one-way ANOVA test) and did not differ from Control uptake (0.33 ± 0.03 Control + CBX).

Taken together these results apparently support an increase in the permeability of Cx43 HCs in parallel with GJs inhibition, upon CKs and LPS treatments. We decided to gain more insights on the role of activated HCs in astrocytes calcium dynamics. To this aim we preincubated Control and treated slices (n = 3 each group) with a distinct Cx43 peptide blocker, namely Gap27 (500 µM; 30 min), known to inhibit HCs opening triggered by chemical or electrical stimuli [44].

**Fig. 5:**
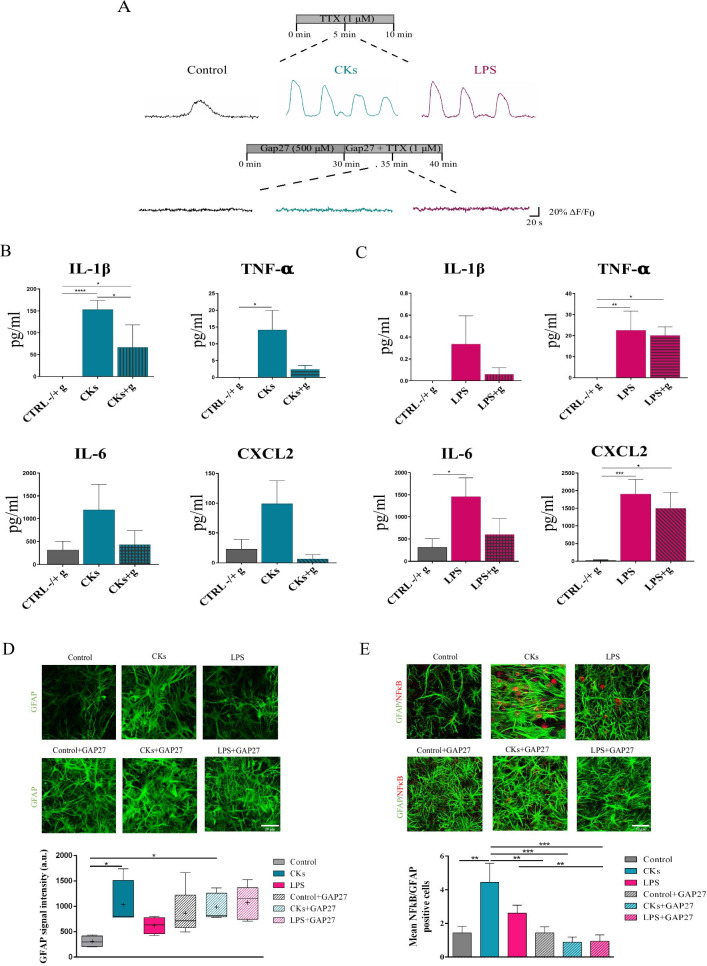
Gap27 removed calcium activity in Control, CKs and LPS astrocytes. **A** Representative glial cells calcium oscillations in Control (black) and after CKs and LPS (blue and purple, respectively). Upon 30 min incubation in Gap27 (500 µM) glial cells activity was completely removed in all conditions. All were performed in TTX. **B** Production of cytokines (IL-1β; TNF-α; IL-6) and chemokine (CXCL2) determined by Milliplex assay of organotypic culture supernatants in CKs prior and after incubation with Gap27 (CKs + g). **C** Production of cytokines (IL-1β; TNF-α; IL-6) and chemokine (CXCL2) determined by Milliplex assay of organotypic culture supernatants in LPS prior and after incubation with Gap27 (LPS + g). Test *P < 0.05, **P < 0.01, ***P < 0.001, ****P < 0.0001, one-way ANOVA. **D** Representative high magnification (60 ×) confocal micrographs of organotypic slices immunolabeled with GFAP (in green) in Control, CKs and LPS prior before (top) or after (bottom) Gap27. GFAP intensity in all conditions is summarized in the box plot. *P < 0.05, Kruskal–Wallis test. **E** Representative high magnification (60 ×) confocal micrographs of organotypic slices co-immunolabeled with GFAP (in green) and NFκB p65 (in red) in Control, CKs and LPS prior (top) or after (bottom) Gap27. The bar plot summarizes the mean number of GFAP/NFκB p65 positive cells in all conditions. **P < 0.01, ***P < 0.001; one-way ANOVA and Mann–Whitney non parametric t-test

In TTX, upon Gap27 treatment, we did not observe any residual calcium activity in astrocytes in untreated or treated cultures (Fig. 5A). This result was strengthened by a separate set of experiments where CBX incubation also removed all calcium activity, thus recapitulating the findings of Gap27 (Additional file 3: Fig. S3; n = 4 each group). Thus, activated HCs are instrumental to the enhanced activity upon inflammation. To explore whether the increased calcium signaling was correlated to astrocyte reactivity and contributed to their inflammatory status we evaluated cytokines and chemokines produced in organotypic cultures after CKs (n = 6) or LPS (n = 6) stimulation [11, 13], compared to Control (n = 6), in the presence or not of the blocking factor Gap27 (“g” in Fig. 5B and C). Overall, in the absence of Gap27, the level of the analyzed soluble factors tends to increase after either CKs and LPS stimulation, compared to Control, confirming our previous observations [11]: this difference is statistically significant in IL-1β CKs-stimulated (P < 0.0001; Fig. 5B), TNFα CKs and LPS-stimulated (P = 0.014 and 0.003, respectively; Fig. 5B and C), IL6 and CXCL2 LPS-stimulated (P = 0.033 and 0.0002, respectively; Fig. 5C). Furthermore, our results show a trend towards a reduction of several cytokines and chemokines in the presence of Gap27, particularly after CKs + g stimulation. These differences become statistically significant in IL-1β, that decreases in CKs + g sample group compared to CKs alone (P = 0.018) and to Control groups (P = 0.029) (Fig. 5B); conversely, TNF-α (P = 0.016; Fig. 5B) and CXCL2 (P = 0.010; Fig. 5C) were still significantly increased in LPS + g group compared to controls, although to a lesser extent when compared to LPS alone. The release of soluble factors is activated by different pathways in CKs or LPS paradigms, thus the extent of cytokines and chemokines detected might be variably tuned by Gap27. We directly investigated whether Gap27 affected astrocyte reactivity induced by inflammatory threads (shown in Additional file 1: Fig. S1D and Fig. 5D top panels and box plot). Figure 5D fluorescence micrographs show that Gap27 (bottom panels) increased GFAP intensity in Control per se, which then remained unmodified in CKs and LPS in the presence of this blocking peptide (box plots in Fig. 5D; n = 4 each condition). This increase in GFAP intensity brought about by Gap27 was apparently unrelated to the inflammatory status but it prevents to fully clarify the relationship between Gap27, GFAP and astrocyte reactivity. To gain more insight on the nature of reactive astrocytes, we addressed the impact of Gap27 on the NFkB (nuclear factor kappa-light-chain-enhancer of activated B cells) pathway, which is strongly associated with neuroinflammation [45–47] and neuroinflammatory reactive astrocytes signaling [48, 49]. Figure 5E shows representative confocal micrographs of GFAP positive astrocytes marked for NFkB prior (top panels) and after (bottom panels) Gap27 incubation. In Control we rarely detected GFAP + astrocytes positive also for NFkB, which were instead present in all CKs and LPS fields (bar plot in Fig. 5E; n = 8 each condition; mean number of double positive cells/condition 1.44 ± 0.40 Control; 4.45 ± 1.12 CKs; 2.61 ± 0.46 LPS; **P = 0.0047 Control vs CKs, one-way ANOVA). In both CKs and LPS, double positive astrocytes were reduced by Gap27 (bar plot Fig. 5E; n = 8 each condition; mean number of double positive cells/condition 0.88 ± 0.34 CKs + GAP27; 0.94 ± 0.37 LPS + GAP27; ***P = 0.0004 CKs vs CKs + GAP27, **P = 0.0020 LPS vs LPS + GAP27; ***P = 0.0005 CKs vs LPS + GAP27; one-way ANOVA and Mann–Whitney non-parametric t-test). Thus, removing HC activity via Gap27 in astrocytes is associated to a block of calcium signaling, a reduction in cytokines and chemokines release and an attenuated NFkB pathway, both events being apparently dissociated to GFAP reactivity.

## Discussion

In the current study we strengthen the role of active HCs in the generation of inflammation-induced calcium dynamics in resident spinal cord astrocytes. We target spinal tissue reactivity, experimentally induced by pro-inflammatory treatments and involving local neuroglia. We imaged, in the spinal organotypic cultures, calcium signaling from glial cells located close to ventral interneurons, identified upon specific labeling [20], displaying calcium episodes resistant to synaptic activity removal and characterized by low pace kinetics [50]. We adopted the organotypic slice model, a well characterized cell system [11–13, 51], where the 3D-architecture of specific resident cells, neuronal and non-neuronal, can be directly investigated after pro-inflammatory treatments [11, 13]. We ignite inflammatory responses by adopting two different acute stresses known to alter synaptic transmission, to trigger different pro- and anti-inflammatory cytokine and chemokines network and to induce different changes in the morphology of GFAP-positive astrocytes and microglia, suggestive of different states of activation [11, 13]. A cytokines cocktail known to exert pro-inflammatory effects in the CNS of multiple sclerosis animal models [4, 11, 13] and LPS, a toll- like receptors (TLR) agonist, in particular of TLR4, expressed on the microglia surface, that influence cytokine production and immune cell function mimicking systemic inflammation [11,13,17]. Despite the induction of alternative activation mechanisms by CKs and LPS [11,13], these two functional conditions similarly enhanced both the number of active glial cells and their oscillatory activity, within the neuronal circuit. We focus on aberrant calcium signals when recorded from astrocytes in the absence of neuronal synaptic activity and restricted to a precise anatomical area, since to understand the functional implications of such signaling requires control of the heterogeneity of reactive cells and of CNS circuits. Apparently, although representing distinct etiologies in the pathology of neuroinflammation, the used danger signals affect glia communication similarly and reactive astrocytes rapidly increase their calcium signals, in frequency and in terms of number of active cells, suggesting that both CKs and LPS promote a similar downstream signaling to translate inflammation into functional changes. It is important to note that we imaged astrocytes within a definite cell layer, and we recorded calcium activity in the cell body located within the focal plan, which prevent monitoring different spatial scales such as astrocyte’s processes. Therefore, we cannot exclude that in CKs as well as in LPS treated slices we analyzed calcium activity limited to a subpopulation of astrocytes with defined phenotypic and functional features [52]. In both inflammatory conditions, aberrant calcium signals occurred spontaneously and relay on Ca^2+^ release from the mitochondria and from the endoplasmic reticulum. In both CKs and LPS treated spinal slices, reactive astrocytes are less synchronized, and indeed WB analysis confirms a reduction in Cx43 protein detection, in accordance with a down regulation of GJs. Our results support the suggestion that Cx43 functions are regulated in an opposite manner by inflammatory stresses, namely with a reduction in GJs channel formation and an increase in permeable un-apposed HCs [39]. The increase cellular uptake of the non-fluorescent permeability tracer EtBr in CKs and LPS is reduced by CBX applications, thus indicating an increased activity of HC upon neuroinflammatory stresses. Indeed, cell death and plasma membrane rupture could influence this dye distribution, however our GFAP counterstaining confirm the presence of intact astrocytes, in addition we have previously shown that both treatments, despite inducing a clear inflammatory reaction, did not affect cell viability [11,13]. HCs role as membrane pathways for signaling mechanisms and diffusion of small molecules or ions (including Ca^2+^) can be regulated by pro-inflammatory CKs [39,53] and further experiments are needed to clarify the relationship among intracellular stores and HCs when contributing to calcium signals. HCs might not be the exclusive membrane pathway contributing to the calcium response, however the use of a mimetic peptide which successfully inhibits abnormal opening of Cx43 HCs [44,54] and displays a better selectivity than general inhibitors such as CBX [55] completely removed astrocyte calcium activity, suggesting that HCs opening is instrumental in such responses or is crucially recruiting other channels to allow gliotransmitters release and the activation of downstream pathways. Indeed, CBX recapitulated these findings. Since both Gap27 and CBX are also GJ blockers [40,41,42], it is not surprising that even in Control calcium activity in astrocytes is removed by these agents.

In line with the hypothesis of HCs opening contributing to inflammation are the downregulation of cytokines and chemokines release upon inflammatory stimuli and the reduction in NFkB positive astrocytes both detected when HCs are blocked. It is relevant to outline that the activation of NFkB pathway is ubiquitous, making it difficult to evaluate its role as essential to initiate astrocyte reactivity. In all conditions, the HCs peptide blocker triggered an increase in GFAP expression, thus it is virtually impossible to assess the role, if any, of HC activation on GFAP increase following CKs or LPS inflammation. This increase in GFAP needs also to be addressed in future investigations, since connexin mimetic peptides have been reported to down regulate GFAP expression, yet after longer treatments [56] when compared to the current experiments. In addition, due to the impairment of astrocytes function due to Gap27, including glial spatial buffering ability of potassium ion excess [57] we cannot exclude that GFAP hypertrophy represent a transient homeostatic response.

Calcium-dependent glia signaling is a highly investigated and well characterized feature of astrocytes, nonetheless its key mechanisms and contribution to inflammatory pathology are still under debate [18, 58]. The finding that in spinal cord circuits local reactive astrocytes display similar calcium signals, but diverse hypertrophy [11, 13] may provide insight into the role of these functional changes. In both the pathological conditions, astrocytes Cx43 HC permeability, key to intracellular signaling including calcium oscillations [59, 60], is enhanced while apparently GJ intercellular trafficking is restricted.

## Conclusions

In this work we confirmed that the organotypic slice system represents an excellent setting, thanks to which it is possible to dissect how the inflammatory environment interferes with the spinal circuits function, due to the easy accessibility of the resident cells and to the long-lasting duration of the cultures. In fact, we exploited these cultures to investigate whole network changes of a precise region of interest under pathological conditions and we were able to mimic two different kinds of inflammation. The main finding of the current work is that local inflammation in organotypic spinal slices, induced by CKs and LPS, was able to regulate the astrocyte function, acting at the calcium signaling level, modulating the permeability of the HCs and altering the GJs communication, regardless GFAP hypertrophy. In conclusion, expanding our knowledge on the interplay between astrocytes and neurons in the spinal circuits and understanding the role of the occurring Ca^2+^ oscillations during pathological conditions represent the starting point for developing new therapies and strategies, essential for investigating the spinal neurodegenerative disorders.

## Methods

### Organotypic spinal cord cultures and pro-inflammatory treatments

All experiments were performed in accordance with the EU guidelines (2010/63/UE) and Italian law (Decree 26/14) and were approved by the local authority veterinary service and by our institution (SISSA) animal wellbeing committee (OBPA). All efforts were made to minimize animal suffering and to reduce the number of animals used. Animal use was approved by the Italian Ministry of Health (no. 22DABNQYA), in agreement with the EU Recommendation 2007/526/CE. Organotypic spinal cord and DRG slices were obtained from mouse embryos (C57BL/6 J) at E12–13 of gestation as previously described [11–13]. Briefly, pregnant mice were sacrificed by CO_2_ overdose and fetuses delivered by caesarean section. Isolated fetuses were decapitated and their backs were isolated from low thoracic and high lumbar regions and transversely sliced (275 μm) with a tissue chopper. After dissecting the spinal cord and the DRG from the surrounding tissue, slices were embedded into a thick matrix obtained by chicken plasma (Sigma) and thrombin (Sigma) clot. Slices were cultured in plastic tubes with 1 mL medium. The tubes were kept in a roller drum rotating 120 times per hour in an incubator at 37 °C in the presence of humidified atmosphere, with 5% CO_2_. Experiments were performed on spinal cultures at 2–3 weeks in vitro (WIV) treated for 6 h with two different inflammatory paradigms [11, 13]: (i) a cocktail of the mouse recombinant cytokines (10 ng/mL each) TNF-α (R&D Systems, #210-TA/CF), IL-1β (R&D Systems, #M15330), and granulocyte–macrophage colony stimulating factor (GM-CSF; R&D Systems, #P04141) [1, 11, 13]; (ii) lipopolysaccharide (LPS; 1 μg/mL, Sigma, O55:B5) [11]. CKs or LPS were removed after the incubation times, prior to live cell imaging recordings, immunoblotting analysis and immunostaining analysis.

### Lentiviruses preparations and organotypic slices infection

The lentiviruses were generated and titrated as previously described [62]. Lentiviral vectors employed in this study include: LV_pGfap-rtTA2S-M2, (built as previously shown [63]) and LV_TREt-mCherry, kindly provided by professor Antonello Mallamaci's lab. The slices were infected with 5 µL of lentiviral preparation, whose titres were 4 × 106 and 1 × 107 respectively. 2 µg/mL doxycycline were administered every two days (TetON system).

### Live cell Ca^2+^ imaging

Organotypic spinal cord cultures were loaded with 4 µM Fluo-4 AM (Molecular Probes); 11.6 µL of DMSO (Sigma-Aldrich) were added to the stock 50 µg of the dye and cultures were incubated with a final concentration of 4 µM for 1 h in the roller drum at 37 °C, 5% CO_2_. After dye loading, a de-esterification period followed, cultures were maintained in extracellular saline solution, also used as recording solution, composed of (mM): 150 NaCl, 4 KCl, 1 MgCl_2_, 2 CaCl_2_, 10 HEPES, 10 Glucose (pH adjusted to 7.35 with 2 M NaOH), in the same incubator for 30 min. The samples were mounted in a recording chamber placed on an inverted microscope (Nikon Eclipse Ti-U), where they were continuously perfused (5 mL/minute flow rate) at room temperature (RT) with the recording saline solution. The dye was excited at 488 nm with a mercury lamp and the emission was detected at 520 nm. Neurons and glial cells (the focus was set and maintained in a slice layer where both neurons and glial cells could be detected) at the premotor region in the ventral zone of the slice were observed with a 40× objective (PlanFluor, 0.60 NA) [23]. Images were constantly acquired at 6.67 fps every 150 ms using an ORCA-Flash4.0 V2 sCMOS camera (Hamamatsu) and the set-up was controlled by HCImage Live software. Basal activity was recorded for 10 min in the presence of saline solution to check for stability prior to adding the following drugs: 1 µM tetrodotoxin (TTX, fast voltage-gated Na^+^ channel blocker, Latoxan); 2 µM carbonyl cyanide 3-chlorophenylhydrazone (CCCP, mitochondrial protonophore, Sigma); 5 µM thapsigargin (TG, SERCA inhibitor, Sigma); 200 µM carbenoxolone (CBX, gap junction uncoupler, Sigma). The kinetics of calcium events, to identify fast (neuronal) and slow (glial) events, was estimated by measuring the episode duration from n = 14 neurons and n = 42 glial cells in 3 cultures. For the Connexin43 mimetic Gap27 (500 µM) experiments, this peptide was dissolved in the saline solution and incubated for 30 min prior to recordings. The recorded images were analyzed selecting ROIs around dye positive cells with Fiji software. The corresponding traces were extracted with Clampfit software (pClamp suite, 10.6 version; Axon Instruments) and analyzed off-line. Ca^2+^ transients were expressed as ΔF/F_0_, where ΔF is the fluorescence rise over baseline, and F_0_ is the baseline fluorescence level, calculated as:

$$100 \times \frac{F-{F}_{0}}{{F}_{0}}$$

(F, fluorescence value; F_0_, baseline fluorescence). F_0_ was calculated as the median of the frame fluorescence values.

### Sulforhodamine 101 (SR101) staining protocol

In order to confirm that the Ca^2+^ activity detected in the presence of TTX was the one of glial cells and in particular of astrocytes, the slices were incubated for 20 min in the recording solution containing 1 µM SR101 at 37 °C, after the incubation with the calcium dye. The slices were then washed with the recording solution for 10 min at 37 °C, to allow the washout of excess dye from the extracellular space. The dye was excited at 594 nm with a mercury lamp.

### Calcium imaging with GCaMP6f and analysis

pZac2.1 gfaABC1D-cyto-GCaMP6f was a gift from Baljit Khakh (Addgene viral prep # 52925-AAV5; http://n2t.net/addgene:52925; RRID:Addgene_52925). Organotypic slices at 1 WIV (n = 4) were infected with AAV5.gfaABC1D-cyto-GCaMP6f which leads to a selective expression of the genetically encoded calcium indicator GCaMP6f in astrocytes. Slices were placed at 2–3 WIV in a custom 3D-printed perfusion chamber mounted on a Nikon microscope (Nikon Eclipse Ti2 microscope endowed with a Nikon IntensiLight Hg lamp and an Andor Zyla sCMOS camera). GCaMP6f fluorescence was recorded with a 20 × S Plan Fluor ELWD NA 0.45 objective once an area within the ventral horn was identified that displayed fluorescence transient activity recognizable by eye. Images were taken at a rate of 1 per 150 ms, with an exposure time of 100 ms and 4 × 4 binning. The acquired time series of images were analysed in Fiji choosing ROIs of ~ Ø 20–30 µm and the obtained mean fluorescence traces were processed in ClampFit 10.7.

### Western blotting analysis

Control and treated organotypic spinal cultures (n = 4 slices per condition) were scraped in 200 µL of Lysis buffer (10 mM Tris–HCl, 150 mM NaCl, 0.5% NP40, 0.5% DOC, protease inhibitor cocktail). Samples were triturated using 200-μL pipette and 1-mL syringe pass tissue suspension through a 26-gauge needle until all tissue were lysed. Subsequently lysates were subjected to 3 freeze–thaw cycles of 1 min each at – 80 °C, sonicated at 50% amplitude for 30 s and centrifuged at 100 × g at 4 °C for 5 min. Protein concentration of the lysate were determined using the bicinchoninic acid assay (Thermofisher Scientific). Samples were prepared adding 2 × Leammli buffer (10% SDS, 20% glycerol, 125 mM Tris–HCl, 0.01% bromophenol blue, 1 M DTT) to 20 μg of proteins and denatured boiling at 100 °C for 5 min. SDS-PAGE gels were prepared in relation to the molecular weight of the protein of interest (12% polyacrylamide separating gel). Samples were run at 120 V at RT and transferred onto Immun-Blot PVDF (Polyvinylidene difluoride) membrane (Millipore) by electroblotting at 100 V for 1 h and 30 min at 4 °C. Membranes were blocked in 5% BSA in TBS-T for 1 h and incubated overnight at 4 °C with anti-Cx43 (rabbit monoclonal, 1:8000, Abcam). The day after, the primary antibody was recovered, the washed membranes in TBS-T were incubated with secondary antibody (Alexa goat anti-rabbit horseradish peroxidase-conjugated, 1:1000, Invitrogen) at RT for 1 h and washed again. As housekeeping protein normalizer was used anti-β-Tubulin III (mouse monoclonal, 1:1000, Sigma) conjugated with the proper secondary antibody (Alexa goat anti-mouse horseradish peroxidase-conjugated, 1:1000, Invitrogen) at RT for 1 h. Subsequently, the membranes were washed with TBS-T and developed by enhanced chemiluminescence (ECL Western Blotting Substrate, Thermofisher) using the UViTEC Cambridge system. The quantified band intensity of three replicates were analyzed using Uviband Analysis, Image quantification Software.

### Immunofluorescence imaging and analysis

For the tissue reactivity analysis, organotypic cultures were fixed with 4% formaldehyde (prepared from fresh paraformaldehyde; Sigma) in PBS (1 ×) for 1 h (RT) and washed in PBS. Free aldehyde groups were quenched in sodium borohydride (NaBH_4_, Sigma) 1% in PBS for 5 min. Slices were permeabilized and blocked in PBS 1 × , 5% FBS (Sigma), 1% BSA (Sigma), and 0.3% Triton X-100 (Sigma) at RT for 1 h and incubated overnight at 4 °C with anti-GFAP (mouse monoclonal, 1:400, Sigma), anti-Iba1 (rabbit polyclonal, 1:500, Wako) and anti-NFκB p65 (rabbit polyclonal, 1:500, Invitrogen) primary antibodies. Subsequently, the slices were PBS-washed and incubated with secondary antibodies diluted in blocking solution for 2 h (RT) in the dark. The secondary antibodies were Alexa 488 goat anti-mouse (1:500, Invitrogen), Alexa 594 goat anti-rabbit (1:500, Invitrogen) and we used DAPI (1:500, Thermo Fisher Scientific) to stain the nuclei. Samples were mounted on glass coverslips using Fluoromount-G aqueous mounting medium (Thermo Fisher Scientific). Images were acquired using Nikon A1 Confocal microscope with Ar/Kr, He/NE, and UV laser with 40 × objective (0.95 N.A.) and 60 × oil objectives (1.35 N.A.) using oil mounting medium (1.515 refractive index). Confocal sections were acquired up to a total Z-stack thickness of 5 to 15 μm (depending on the required analysis) in sequential mode with lasers (488 nm for GFAP and 561 nm for Iba1 and NFκB p65). For each experiment we performed ≥ 3 independent cultures; from each culture series, we used ≥ 3 slices for every condition, and from each slice, ≥ 5 fields were randomly acquired from the ventral region. Offline analysis of the image Z-stack was performed using the Volocity 3D Image Analysis Software. The offline analysis of the Z-stacks for calculating the transformation index and the GFAP signal intensity were performed as previously reported [11]. Briefly, for microglia morphology, we measured the area and the perimeter, necessary to calculate the transformation index [11] as $$\frac{{[perimeter of cell \left(\mu m\right)]}^{2}}{4\pi \bullet [area of cell \left({\mu m}^{2}\right)]}$$ quantifying microglia ramifications. Astrocytes were quantified by measuring, in every acquired field, the intensity of the GFAP signal calculated by the software using a greyscale and expressed in the plot in arbitrary unit (a.u.). To quantify the astrocytes proinflammatory activation we evaluate the expression of the transcription factor NFκB p65 in GFAP positive cells. Double positive cells were identified by the Volocity tool “Intersect objects” to quantify only the objects where NFκB p65, GFAP, and DAPI signals are intersected. Furthermore, a size threshold limit was set to exclude all the objects with a dimension lower than 5 µm^3^ in order to avoid background signal interference. The results were expressed as the mean number of NFκB p65/GFAP positive cells for each treatment condition.

### Ethidium bromide uptake

For dye uptake experiments, cultures were exposed to 5 μM EtBr (Sigma) for 10 min at 37 °C. In order to investigate the contribution of GJs and HCs to the dye uptake, an independent experiment was performed where slices were incubated with CBX for 10–15 min prior to EtBr + CBX for additional 30 min. EtBr is permeable through membrane but can transit through HCs and becomes more fluorescent after binding to DNA. After 30 min exposure to EtBr, the slices were washed in extracellular saline solution for 15 min, fixed in 4% formaldehyde (prepared from fresh PFA, Sigma) in PBS 1 × for 1 h at RT and washed in PBS. Free aldehyde groups were quenched in 0.1 M glycine in PBS for 10 min. Slices were permeabilized and blocked in PBS 1 × , 10% FBS (Sigma), 5% BSA (Sigma), and 0.3% Triton-X 100 (Sigma) at RT for 1 h, and then incubated over night at 4 °C with anti-GFAP primary antibody. Secondary antibodies were Alexa 488 goat anti-mouse (1:500, Invitrogen) and DAPI (1:500, Thermo Fisher Scientific) diluted in blocking solution for 2 h at RT, in the dark. Samples were mounted on glass coverslips using Fluoromount-G aqueous mounting medium (Thermo Fisher Scientific). Images were acquired using Nikon C2 Confocal microscopes with Ar/Kr, He/NE, and UV laser with 40 × oil objectives (1.4 N.A.) using oil mounting medium (1.515 refractive index). Confocal sections were acquired every 0.5 μm up to a total *Z*-stack thickness of 20 μm in sequential mode with lasers (488 nm for GFAP and 561 nm for EtBr). For each condition, we performed 3 independent cultures (3 slices/series), and from each slice ≥ 5 fields were randomly acquired. Offline analysis of the image Z-stack was performed using the Volocity3D Image Analysis Software. The “GFAP^+^ objects” and the “EtBr^+^ objects” were determined after thresholding images. With the Volocity tool “Intersect objects” we determined the number of “EtBr^+^ astrocytes”. EtBr uptake was expressed as the ratio between the “EtBr^+^ astrocytes” over the total number of astrocytes (“GFAP^+^ objects”).

### Cytokines and chemokines: Luminex assay and analysis

In organotypic culture supernatants 13 cytokines and chemokines were measured (IFN-γ, IL-1α, IL-1β, IL4, IL6, IL10, IL12p40, IL12p70, IL17, CXCL10, CCL2, CXCL2, TNF- α) by Milliplex assay (Merck Millipore, USA, #MCYTOMAG-70 K) using Bio-Plex device (Bio-Rad, USA), according to the manufacturer’s protocol. For all the analyzed factors, the assay detection limit was below 1.1 pg/ml. Statistical analysis was performed by One-Way ANOVA followed by post-hoc Tukey's multiple comparisons test. Significance was considered when P < 0.05.

### Statistical analysis and analysis of synchronization

All values from samples subjected to the same experimental protocols were pooled together and results are presented as mean ± S.E.M. with n = number of slices, if not stated otherwise. A statistically significant difference between three data sets was assessed by one-way ANOVA for parametric data or Kruskal–Wallis test for non-parametric ones and Mann–Whitney non parametric t-test. In addition, differences in the relative cumulative frequency distribution were obtained using the paired Kolmogorov–Smirnov test. Statistical significance was determined at P < 0.05.

In box-plots, the thick horizontal bar indicates the median value, the cross indicates the mean value, the boxed area extends from the 25^th^ to 75^th^ percentiles while whiskers from the 5^th^ to the 95^th^ percentiles.

The correlation between the oscillatory activities of two neighbor cells in the same slice was assessed by cross correlation analysis. The synchronization analysis was based on a bootstrapping method modified from Usmani et al. [64]. With this analysis, 200.000—time windows are generated from each pair of traces and used to obtain a “real CCF (cross-correlation factor)” distribution with its mean/median, then compared with the distribution of “randomly generated CCFs” obtained by shuffling the 200.000—time windows. The extent of the area of the “random CCFs” distribution that exceeded the mean/median of the “real CCFs” allowed the calculation of a p-value. Significantly (*P < 0.05) correlated pairs were considered synchronized and their count over the total number of cell pairs analyzed was plotted as percentage of correlated pairs. The distribution of the *p*-values was also plotted. For estimating significantly synchronous slices in the three groups, we performed a homogeneity test with the Fisher’s exact test.

More in detail, we measured three slices per experimental condition and we obtained the measurements from non-overlapping neurons or glial cells. Here, we found that paired neurons (n = 135, 135 and 156 pairs in Control, CKs and LPS) in all the three different experimental conditions are synchronized (0.004 ± 0.001 pair *P*-value Control; 0.001 ± 0.0002 pair *P*-value CKs 6H; 0.001 ± 0.0002 pair *P*-value LPS 6H, Kruskal–Wallis test and Fisher’s exact test; Additional file 2: Fig. S2), with no significant differences among the groups.

## Supplementary Information

**Additional file 1: Figure S1. **Pro-inflammatory treatments boost neuronal calcium signaling and alter glial reactivity. A. Left, GCaMP6f fluorescence image, maximal projection of 3554 images acquired in 10 min of recording at 6.6 fps. Scale bar, 50 μm. Right, representative GCaMP6f fluorescence recordings from Control organotypic ventral horn before (in black) and after TTX (1 μM, in blue) application and (right) results are pooled together in the boxplot and cumulative distribution of interevent intervals (IEI, n_Control_ = 32 cells and n_TTX_ = 46 cells, P = 0.4539). B. Representative snapshots (40 × magnification) of the ventral area of organotypic spinal slices loaded with Fluo-4 AM; frames were taken at variable time intervals (0, 5 and 10 s) in three different experimental conditions (Control, CKs and LPS). Scale bar 50 µm. C. Representative fluorescent tracings depicting neuronal spontaneous activity as calcium transients in control (black) and after CKs and LPS (blue and purple, respectively). The box plot summarizes the frequency values of calcium events in all conditions. ***P < 0.001 and **P < 0.01, Kruskal–Wallis. D. Representative confocal images of organotypic spinal slices immunolabeled for Iba1 (red) and GFAP (green), visualizing microglia and astrocytes, respectively, prior and after CKs or LPS administration. Scale bar 25 μm. E. Box plot (top) summarizes GFAP signal intensity prior and after CKs or LPS treatments. Test ****P < 0.0001 Control vs CKs, ***P = 0.001 CKs vs LPS, P = 0.0112 Control vs LPS, one-way ANOVA. Box plot (bottom) summarizes transformation indices upon CKs and LPS administration. *P < 0.05 Control vs CKs and ****P < 0.0001 Control vs LPS, Kruskal–Wallis test.

**Additional file 2: Figure S2. **Pro-inflammatory treatments alter glial cells synchronization. A. Two example fluorescent tracings, obtained from two neurons (in red and blue) located in the same visual field in the ventral horn of organotypic slices in Control, CKs and LPS. The fluorescent recordings show calcium oscillations and the synchrony between recorded neurons was determined by computing their Pearson correlation coefficient in time windows that were randomly sampled from the all duration of the recording. The bar plot shows the % of correlated pairs in Control, CKs and LPS. Kruskal–Wallis test. The aligned dot plot shows the p values distributions obtained by the comparison of correlated pairs of traces. Fisher’s exact test. B. Two example fluorescent tracings, obtained from two astrocytes (in red and blue) located in the same visual field in the ventral horn of organotypic slices in Control, CKs and LPS. The fluorescent recordings show calcium oscillations in the presence of TTX and the synchrony between recorded astrocytes was determined by computing their Pearson correlation coefficient in time windows that were randomly sampled from the all duration of the recording. The bar plot summarizes the % of correlated pairs detected in all conditions. ***P < 0.001 and **P < 0.01, one-way ANOVA. The aligned dot plot (right) shows the p values distribution obtained by the comparison of correlated pairs of traces. *p < 0.05 Fisher’s exact.

**Additional file 3: Figure S3. **CBX removed calcium activity in Control, CKs and LPS astrocytes. A. The scatter plot shows the mean number of active glial cells/ slices in Control, CKs and LPS (in black, cyan and purple, respectively), before and after the administration of carbenoxolone (CBX, 200 µM). Each dot in the plot represents one different slice. B. Representative fluorescent tracings of glial cells calcium events, prior and after CBX, in Control (black) and after CKs and LPS administration (cyan and purple, respectively).

## Data Availability

The datasets supporting the conclusion of this article are included within the article (and its additional files). The datasets generated and/or analysed during the current study are stored in a public repository and are available from the corresponding author on reasonable request.

## Abbreviations

ALS: Amyotrophic lateral sclerosis
CBX: Carbenoxolone
CCCP: 2 Carbonyl cyanide 3-chlorophenylhydrazone
CCF: Cross correlation coefficient
CKs: Cytokines
CNS: Central nervous system
Cx43: Connexin 43
DRG: Dorsal root ganglia
ER: Endoplasmic reticulum
EtBr: Ethidium bromide
GM-CSF: Granulocyte–macrophage colony-stimulating factor
GJs: Gap junctions
HCs: Hemichannels
IL-1β: Interleukin 1 beta
LPS: Lipopolysaccharide
MS: Multiple sclerosis
RT: Room temperature
SERCA: Sarco-endoplasmic reticulum
SR101: Sulforhodamine 101
TG: Thapsigargin
TLR: Toll-like receptor
TNF-α: Tumor necrosis factor alpha
TTX: Tetrodotoxin
WIV: Weeks in vitro
WB: Western blot
