# Supplementary figures and images for: Diverse inflammatory threats modulate astrocytes Ca2+ signaling via connexin43 hemichannels in organotypic spinal slices
Source: Mol Brain. 2021 Oct 25;14:159. doi: 10.1186/s13041-021-00868-6 (PMC8547100; doi:10.1186/s13041-021-00868-6)

A

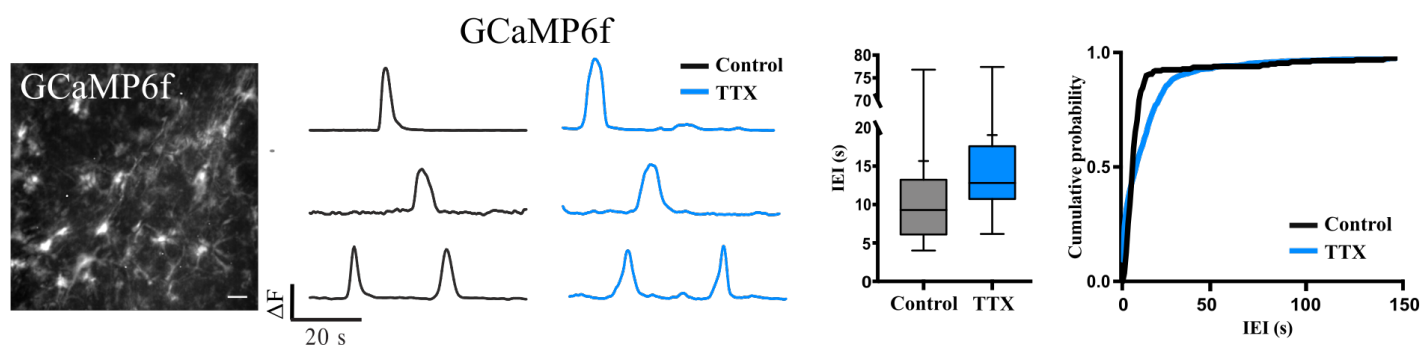

B

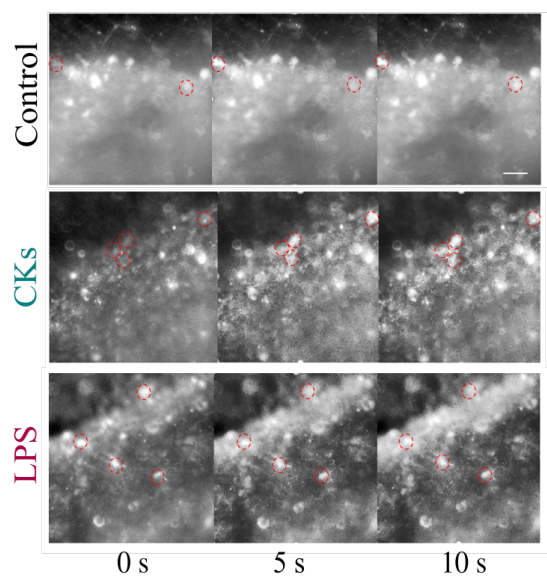

C

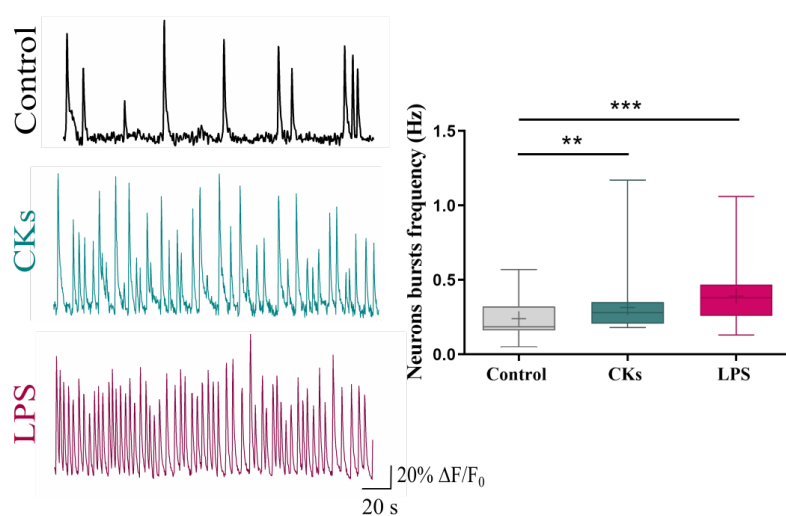

D

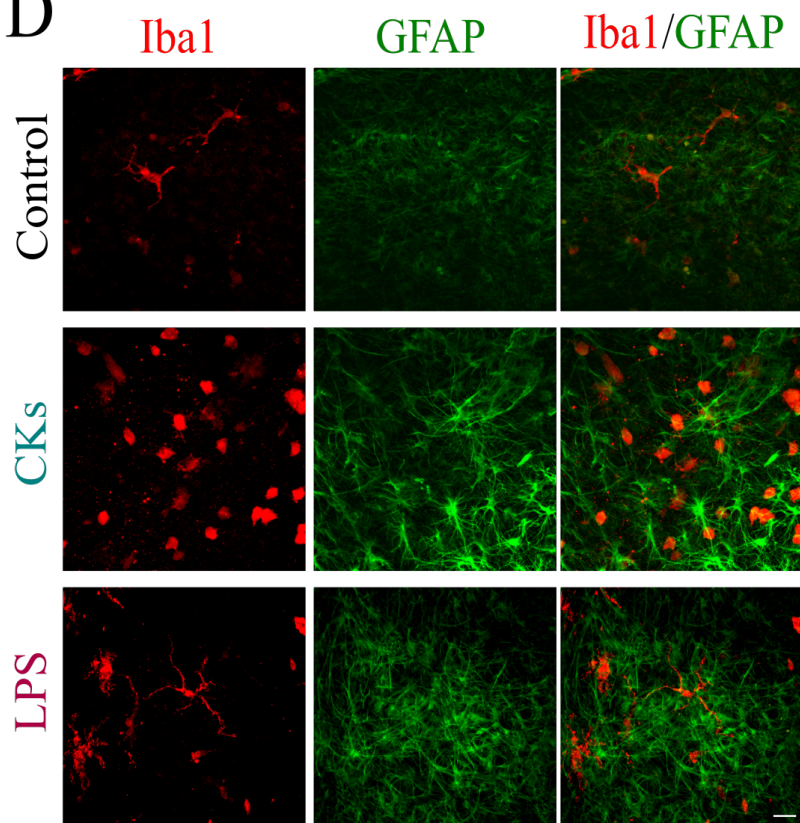

E

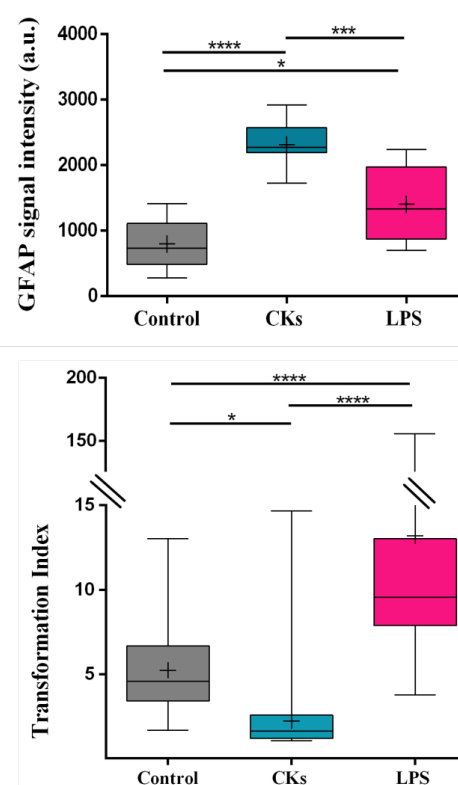

Supplement: Supplementary file 1 — Additional file 1: Figure S1. Pro-inflammatory treatments boost neuronal calcium signaling and alter glial reactivity. A. Left, GCaMP6f fluorescence image, maximal projection of 3554 images acquired in 10 min of recording at 6.6 fps. Scale bar, 50 μm. Right, representative GCaMP6f fluorescence recordings from Control organotypic ventral horn before (in black) and after TTX (1 μM, in blue) application and (right) results are pooled together in the boxplot and cumulative distribution of interevent intervals (IEI, nControl = 32 cells and nTTX = 46 cells, P = 0.4539). B. Representative snapshots (40 × magnification) of the ventral area of organotypic spinal slices loaded with Fluo-4 AM; frames were taken at variable time intervals (0, 5 and 10 s) in three different experimental conditions (Control, CKs and LPS). Scale bar 50 µm. C. Representative fluorescent tracings depicting neuronal spontaneous activity as calcium transients in control (black) and after CKs and LPS (blue and purple, respectively). The box plot summarizes the frequency values of calcium events in all conditions. ***P < 0.001 and **P < 0.01, Kruskal–Wallis. D. Representative confocal images of organotypic spinal slices immunolabeled for Iba1 (red) and GFAP (green), visualizing microglia and astrocytes, respectively, prior and after CKs or LPS administration. Scale bar 25 μm. E. Box plot (top) summarizes GFAP signal intensity prior and after CKs or LPS treatments. Test ****P < 0.0001 Control vs CKs, ***P = 0.001 CKs vs LPS, P = 0.0112 Control vs LPS, one-way ANOVA. Box plot (bottom) summarizes transformation indices upon CKs and LPS administration. *P < 0.05 Control vs CKs and ****P < 0.0001 Control vs LPS, Kruskal–Wallis test. [file 13041_2021_868_MOESM1_ESM.pdf]

A

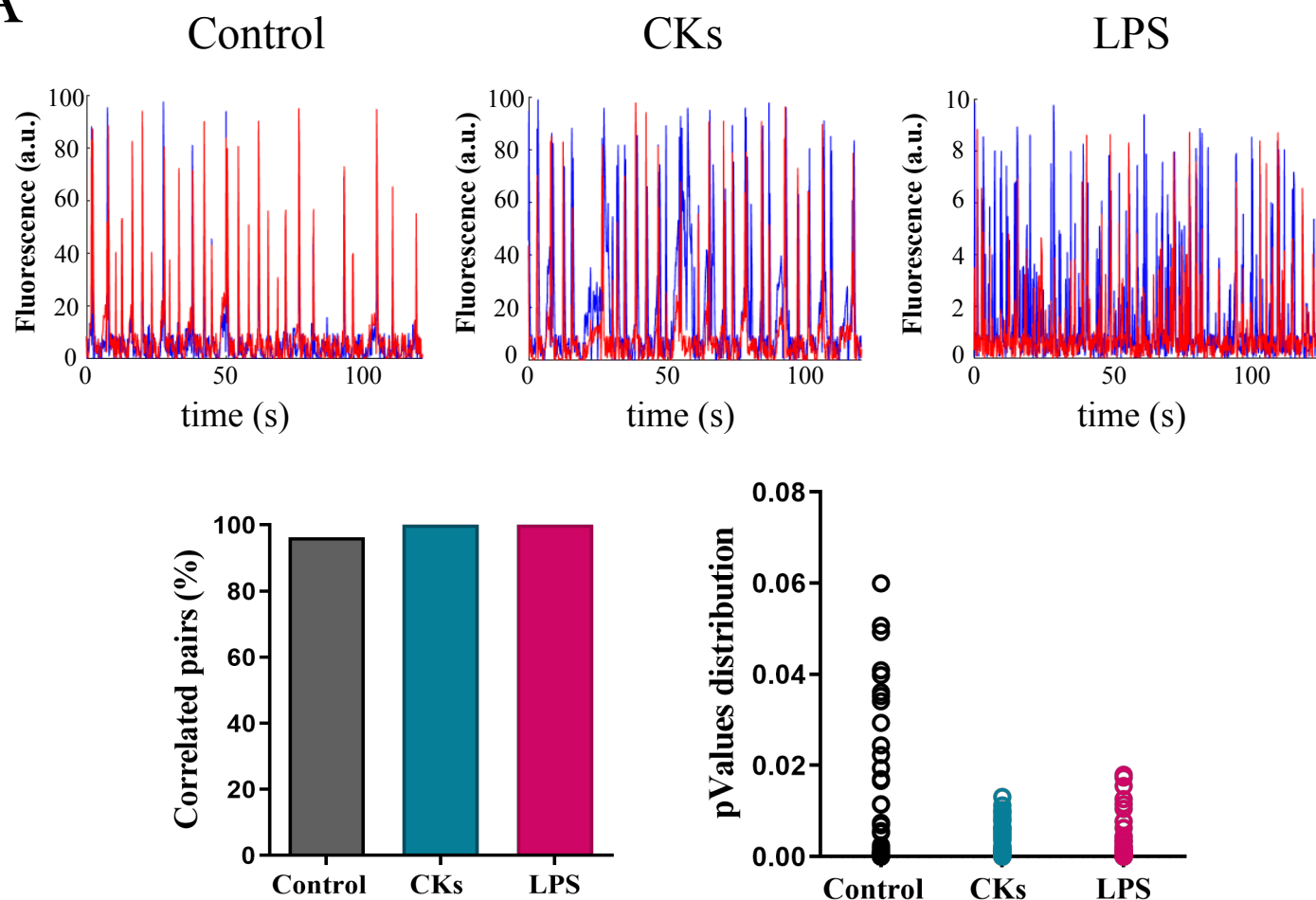

B

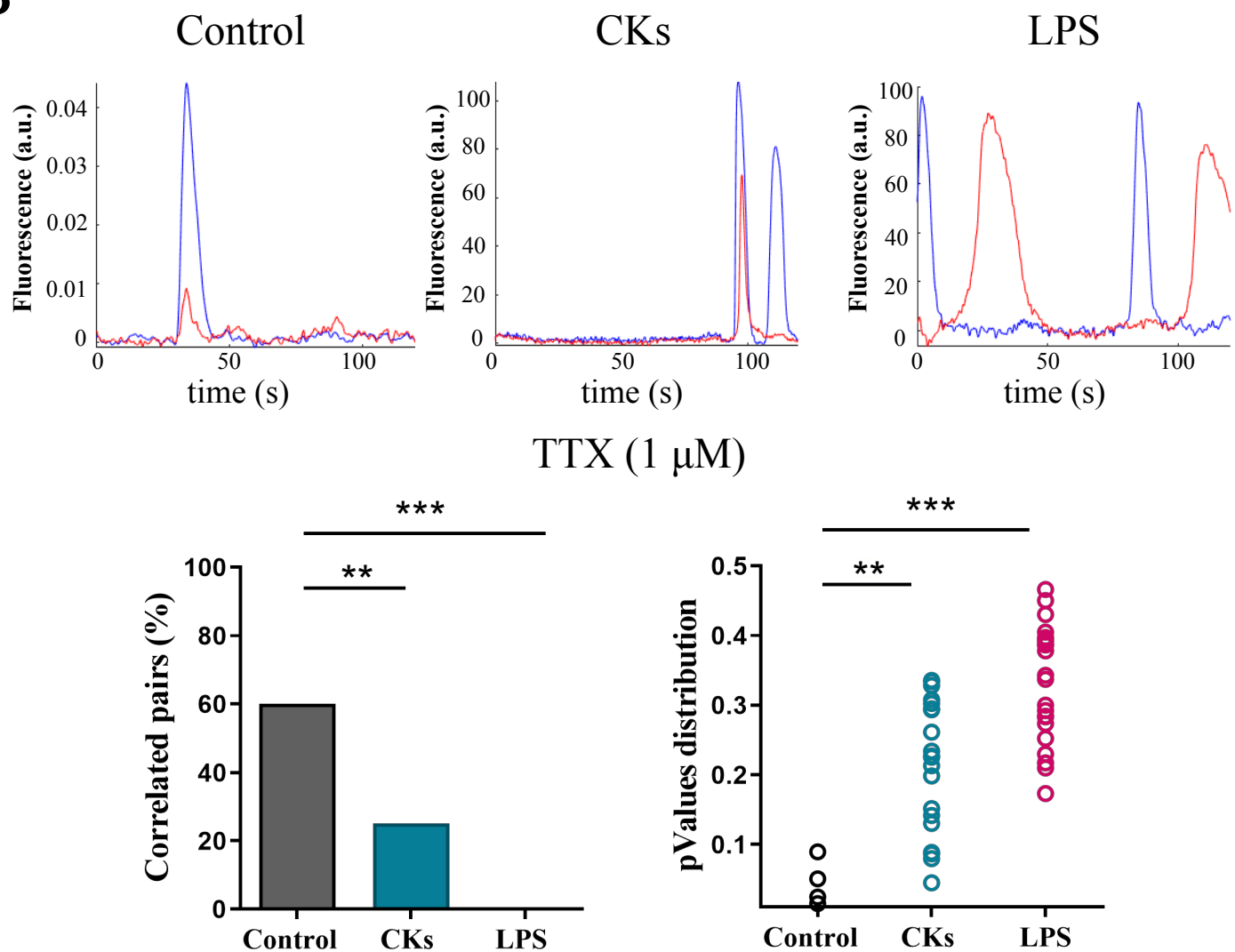

Supplement: Supplementary file 2 — Additional file 2: Figure S2. Pro-inflammatory treatments alter glial cells synchronization. A. Two example fluorescent tracings, obtained from two neurons (in red and blue) located in the same visual field in the ventral horn of organotypic slices in Control, CKs and LPS. The fluorescent recordings show calcium oscillations and the synchrony between recorded neurons was determined by computing their Pearson correlation coefficient in time windows that were randomly sampled from the all duration of the recording. The bar plot shows the % of correlated pairs in Control, CKs and LPS. Kruskal–Wallis test. The aligned dot plot shows the p values distributions obtained by the comparison of correlated pairs of traces. Fisher’s exact test. B. Two example fluorescent tracings, obtained from two astrocytes (in red and blue) located in the same visual field in the ventral horn of organotypic slices in Control, CKs and LPS. The fluorescent recordings show calcium oscillations in the presence of TTX and the synchrony between recorded astrocytes was determined by computing their Pearson correlation coefficient in time windows that were randomly sampled from the all duration of the recording. The bar plot summarizes the % of correlated pairs detected in all conditions. ***P < 0.001 and **P < 0.01, one-way ANOVA. The aligned dot plot (right) shows the p values distribution obtained by the comparison of correlated pairs of traces. *p < 0.05 Fisher’s exact. [file 13041_2021_868_MOESM2_ESM.pdf]

# A

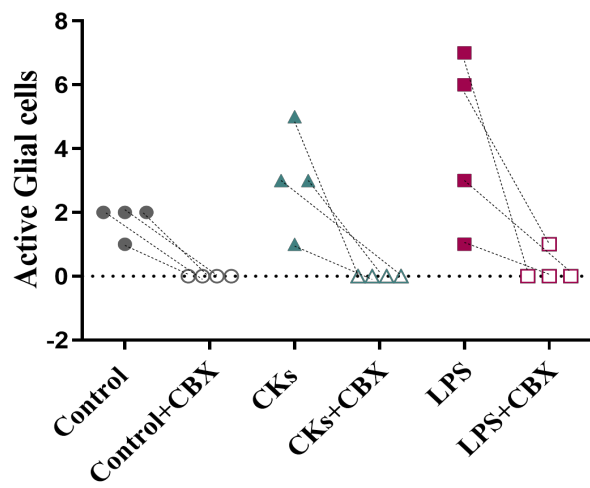

# B

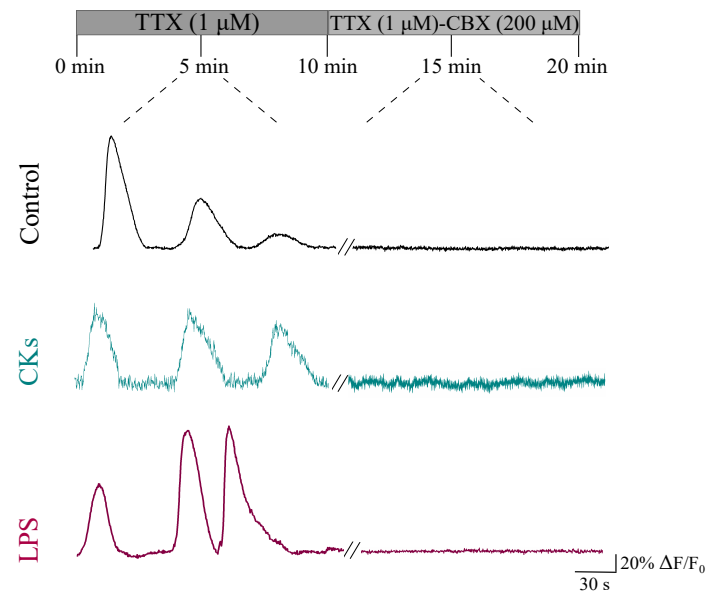

Supplement: Supplementary file 3 — Additional file 3: Figure S3. CBX removed calcium activity in Control, CKs and LPS astrocytes. A. The scatter plot shows the mean number of active glial cells/ slices in Control, CKs and LPS (in black, cyan and purple, respectively), before and after the administration of carbenoxolone (CBX, 200 µM). Each dot in the plot represents one different slice. B. Representative fluorescent tracings of glial cells calcium events, prior and after CBX, in Control (black) and after CKs and LPS administration (cyan and purple, respectively). [file 13041_2021_868_MOESM3_ESM.pdf]
